# The Decrease of Uch-L1 Activity Is a Common Mechanism Responsible for Aβ 42 Accumulation in Alzheimer’s and Vascular Disease

**DOI:** 10.3389/fnagi.2017.00320

**Published:** 2017-09-29

**Authors:** Michela Guglielmotto, Debora Monteleone, Valeria Vasciaveo, Ivan Enrico Repetto, Giusi Manassero, Massimo Tabaton, Elena Tamagno

**Affiliations:** ^1^Department of Neuroscience, University of Torino, Torino, Italy; ^2^Neuroscience Institute of Cavalieri Ottolenghi Foundation (NICO), University of Torino, Torino, Italy; ^3^Department of Neuroscience, Université de Lausanne, Lausanne, Switzerland; ^4^Department of Internal Medicine and Medical Specialties (DIMI), Unit of Geriatric Medicine, University of Genova, Genova, Italy

**Keywords:** Alzheimer’s disease, amyloid beta, mixed dementia, Uch-L1, BACE1

## Abstract

Alzheimer’s disease (AD) is a multifactorial pathology causing common brain spectrum disorders in affected patients. These mixed neurological disorders not only include structural AD brain changes but also cerebrovascular lesions. The main aim of the present issue is to find the factors shared by the two pathologies. The decrease of ubiquitin C-terminal hydrolase L1 (Uch-L1), a major neuronal enzyme involved in the elimination of misfolded proteins, was observed in ischemic injury as well as in AD, but its role in the pathogenesis of AD is far to be clear. In this study we demonstrated that Uch-L1 inhibition induces BACE1 up-regulation and increases neuronal and apoptotic cell death in control as well as in transgenic AD mouse model subjected to Bengal Rose, a light-sensitive dye inducing that induces a cortical infarction through photo-activation. Under the same conditions we also found a significant activation of NF-κB. Thus, the restoration of Uch-L1 was able to completely prevent both the increase in BACE1 protein levels and the amount of cell death. Our data suggest that the Uch-L1-mediated BACE1 up-regulation could be an important mechanism responsible for Aβ peptides accumulation in vascular injury and indicate that the modulation of the activity of this enzyme could provide new therapeutic strategies in AD.

## Introduction

Although Alzheimer’s disease (AD) is considered a neurodegenerative disease evidence-based pathology and epidemiology studies associate it with a vascular disease (Grammas et al., 2011).

Thus, vascular damage is often present in patients diagnosed with AD and mixed dementia and it is significant factor in 10%–20% of cases (Schneider et al., 2007; Kapasi and Schneider, 2016). Epidemiological evidence also suggests that mixed AD and vascular diseases are reported to be common in older adults where AD and vascular dementia share symptomatic, pathological and neurochemical characteristics (Santos et al., 2017). Some authors have recently developed the “vascular hypothesis of AD”, according to which vascular damage would be responsible for neurodegeneration. Studies supporting this theory show that vascular damage induces inefficient cerebral clearance of Aβ, causing its accumulation in the parenchyma and blood vessel (Snyder et al., 2015; Janota et al., 2016).

Recently, we showed that Aβ 42 inhibits the activity of ubiquitin C-terminal hydrolase L1 (Uch-L1). This event is related to an up-regulation of BACE1, mediated by the activation of NF-κB pathway as well as by an impairment of its lysosomal degradation (Guglielmotto et al., 2012).

Uch-L1 is a neuronal enzyme representing 1%–2% of the total brain proteins (Wilkinson et al., 1989). Uch-L1 function is to remove ubiquitin from proteins that need to be directed to proteasome pathway (Gong and Leznik, 2007).

It has been suggested that its role is particularly important in removing excess, oxidized or misfolded proteins both in physiology and pathology.

Thus, down-regulation of Uch-L1 induces the aggregation of ubiquitinated proteins and promotes cell death in neurons. The decrease of this enzyme was observed in both ischemic injury and AD (Wang et al., 2017), but the role of this decrease in the pathogenesis of the diseases is far to be clear.

Of note, some authors report that the activity of Uch-L1 is lower in AD brain (Pasinetti, 2001; Choi et al., 2004) and its levels are inversely proportional to the number of neurofibrillary tangles (NFT) in sporadic AD brain patients (Chen et al., 2013). Then, gracile axonal dystrophy mice that did not express Uch-L1 have high levels of Aβ (Ichihara et al., 1995) and in a double transgenic AD mouse model the Uch-L1 activity was found diminished (Gong et al., 2006). Proteomics analysis indicated that the level of Uch-L1 was lower in AD hippocampal proteome (Sultana et al., 2007). Immunohistochemical studies showed that Uch-L1 is associated with NFT and the reduction of soluble Uch-L1 was inversely proportional to the number of NFT in AD brains (Choi et al., 2004; Chen et al., 2013). Moreover, Minjarez et al. (2013) identified that the NFT derived from AD brains contained Uch-L1 component and proved the colocalization of Uch-L1 and hyperphosphorylated Tau protein in NFT.

In this study we demonstrated that the inhibition of Uch-L1 induces BACE1 up-regulation and increases neuronal cell death in control as well as in AD transgenic mouse models subjected to Bengal Rose, a light-sensitive dye inducing a cortical infarction through photo-activation. Our data suggest that the Uch-L1-mediated BACE1 up-regulation could be an important mechanism for Aβ peptides accumulation both in AD and cerebrovascular lesions.

## Materials and Methods

### Animals

Two-month-old no carrier (control mice) and B6SJL-Tg(APPSwFlLon, PSEN1* M146L*L286V)6799Vas/Mmjax (5XFAD Tg mice) were used for producing the focal cerebral ischemia.

Experimental procedures involving the use of live animals have been carried out in accordance with the guidelines established by the European Community Directive 86/609/EEC (November 24, 1986), Italian Ministry of Health and the University of Turin institutional guidelines on animal welfare (law 116/92 on Care and Protection of living animals undergoing experimental or other scientific procedures; authorization number 17/2010-B, June 30, 2010). Moreover, the Ethical Committee of the University of Turin approved this type of studies.

The animals were maintained under 12-h light/dark cycles and were provided with water and food “*ad libitum*” (standard mouse chow 4RF25-GLP, Mucedola srl, Settimo Milanese, Italy). Specifically, all the procedures were carried out in order to minimize the pain and distress in the animals and we used the fewest number of animals required to obtain statistically significant data.

### Photothrombotic Focal Ischemia

Focal ischemia was performed as previously described (Labat-gest and Tomasi, 2013). Briefly, 10 min before surgery, the animals were i.p injected with Rose Bengal (15 mg/ml) according to their body weight (10 μl/g). Then, we used 4% isoflurane (Isoflurane-Vet 100%, Liquid, Merial Italy, Milan, Italy) vaporized in O_2_/N_2_O 50:50 to anesthetize wt and tg mice. The anesthesia was maintained at 1.5%–2.5% isoflurane while mice were in the stereotaxic apparatus for small rodents (Stoelting, Wood Dale, IL, USA). The skull was exposed and an optical fiber cable (150 W) was placed in close contact with a small region (approximately 30 mm^2^) 2 mm lateral to the Bregma, corresponding to the sensory motor cortex (Vogt and Paxinos, 2014). After 15 min of illumination the mice were sutured and placed in a warm cage for recovery.

After 24 h the animals were killed by using an overdose of anesthetic, the brains were collected and cut with a mouse brain matrix in 1 mm coronal slices; brain extracts were removed from lesioned and controlateral areas. Then the samples were prepared for Triphenyl-Tetrazolium Chloride (TTC) staining. The TTC staining was used to stain and allow dissection of the lesioned areas and not to quantify the amount of the ischemic damage. The TTC reaction was stopped with 4% paraformaldehyde (PFA) in 0.1 M phosphate buffer (PB, pH 7.4). The brain slices were maintained in the fixative for 2 weeks and then processed for the isotropic fractionator.

### Isotropic Fractionator Method

Isotropic fractionator was executed according to Herculano-Houzel and Lent (2005) following a TTC staining as described before (Repetto et al., 2016). Briefly, after the neural tissue was properly fixed, a single brain slice per animal was collocated into a glass tissue grinder, a saline-detergent solution consisting of 40 mM sodium citrate and 1% Triton™ X-100 (Sigma-Aldrich, St. Louis, MO, USA) was added and the tissue was carefully homogenizated to obtain a nuclear suspension. The nuclear suspension obtained was stained with the fluorescent DNA dye 4′-6-diamino-2-phenylindole dihydrochloride (DAPI; DAPI, dilactate, D9564, Sigma-Aldrich, St. Louis, MO, USA). Aliquots from the isotropic suspension were loaded into a hemeocytometer (Neubauer chamber) and observed under fluorescence microscopy (Nikon Eclipse 80i). The nuclei density was evaluated by counting the number of nuclei within sectors of the hemeocytometer coverslipped (1 mm^2^ area; 0.1 mm depth) of four aliquots for sample. To recognize the fraction of neuronal nuclei among the total number of DAPI-stained nuclei, another aliquot of the isotropic suspension was collected and stained with mouse primary antibody for the neuronal nuclear protein NeuN (MAB377, Chemicon, Single Oak Drive, Temecula, CA, USA, 1:200 in PBS, overnight incubation at room temperature (RT)). Then, the nuclei were washed in saline and incubated at RT for at least 2 h with the secondary Cy3 conjugated anti-mouse donkey antibody (Chemicon, Single Oak Drive, Temecula, CA, USA; 1:200 in PBS) and normal donkey serum (1:10; D9663, Sigma-Aldrich, St. Louis, MO, USA). The non-neuronal cells were obtained as the difference between the total number of cells and the total number of neurons.

### Expression and Purification of Recombinant TAT Fusion Proteins

TAT-fused Uch-L1 was provided by Dr. Ottavio Arancio (Professor at Columbia University), the construct was obtained as described by Gong et al. (2006).

Briefly, TAT vectors were transformed into *E. Coli* BL21(DE3) pLysS competent cells (Novagen), and the obtained colonies were grown as 1 ml overnight cultures in Luria broth (LB) medium (Sigma-Aldrich) with 100 mg ampicillin, in the presence of 100 mM IPTG. Then the cultures were transferred to 500 ml LB ampicillin plus 200 mM IPTG to obtain large-scale preparations. Fusion proteins were purified according to ProBond purification system (Invitrogen).

VUch-L1 fusion proteins were i.p. injected into mice at 0.03 g/kg, 20 min before the Rose Bengal injection and surgery procedure. After 6 or 12 h, mice were sacrificed and protein extracts were prepared and examined as described below.

### Antibodies and Immunoblot Analyses

The following antibodies were used for immunoblotting analyses: BACE1 (Millipore, AB5940, 1:500), pJNK1/2 (Cell Signaling Technology, #9251, 1:500); JNK1/2 (Cell Signaling Technology, #9252, 1:500); BAX (Santa Cruz Biotechnology, Sc-493, 1:100); Bcl-2 (Santa Cruz Biotechnology, Sc-509, 1:200); β actin (Sigma-Aldrich, A5441, 1:5000); Uch-L1 (Santa Cruz Biotechnology Sc-1183, 1:200).

Fresh frozen brains were homogenized in ice-cold buffer consisting of 20 mM Tris-HCl pH 7.4, 150 mM NaCl, 2 mM EGTA, 1 mM EDTA, 1% Triton™-X-100, 1 mM PMSF, phosphatase and protease inhibitors and then centrifuged at 12,000 rpm for 20 min at 4°C in order to obtain soluble proteins. Lysates (20 μg) were run on 4%–12% Tris-HCl gradient PAGE gel (Invitrogen) and then transferred to nitrocellulose blotting membrane (GE Healthcare 10600008). Peroxidase-conjugated secondary antibodies were incubated 1 h at RT and revealed with Luminata Forte Western substrate (WBLUF0100, Millipore). The correct protein loading was controlled normalizing with β actin antibody.

### Evaluation of Aβ 42 Production by ELISA

Whole cell extracts were made in ice-cold lysis buffer (PBS, TritonX-100, SDS 10%, DTT 1 M, PMSF 0.1% and aprotinin) for 30 min and sonicated for 1 min. The lysates were centrifugated at 17,860 *g* for 15 min to clarify the suspensions. The protein concentration was quantified following Bradford’s method (1976). The amount of Aβ 42 was evaluated using the Human/Rat βAmyloid ELISA Kit (Wako Chemicals GmbH, Neuss, Germany) according to the manufacturer’s instructions.

### BACE1 Activity

BACE-1 activity was measured using a commercially available secretase kit from Calbiochem (Merck, Darmstadt, Germay), according to the manufacturer’s protocol. Briefly, samples were lysed in cold 1× Extraction Buffer (provided by the kit) to obtain a final protein concentration of 1 mg/mL.

The method is based on the ability of the enzyme to cleave a peptide conjugated with a reporter molecules (EDANS and DABCYL). The cleavage induces the release of a fluorescent signal that was detected using a fluorescence microplate reader (excitation wavelength of 355 nm and emission 510 nm) and the signal is proportional to the enzymatic activity. BACE1 activity was expressed as percentage change over activity level of control samples (Guglielmotto et al., 2012).

### Hydrolase Activity Assay

The hydrolase activity assay was performed using the fluorogenic ubiquitin-7-amino-4-methylcoumarin (ubiquitin-AMC; Boston Biochem, Cambridge, MA, USA) substrate diluted in an assay buffer (50 mM Tris–HCl pH 7.6, 0.5 mM EDTA, 5 mM DTT and 0.1 mg mL ovalbumin). The reaction mixture containing 400 nM substrate and 100 μg protein samples was incubated for 5 min at RT and the enzymatic activity was measured using a fluorescence spectrometer (LS55; Perkin Elmer Instruments, Waltham, MA, USA) at 25°C (EX 380 nm and EM460 nm; Guglielmotto et al., 2012).

### NF-κB Activity

The activity of NF-κB was measured using a commercially available kit (Active Motif, Rixensart, Belgium). The NF-κB contained in the nuclear extracts specifically binds to an oligonucleotide containing an NF-κB consensus binding site. The primary antibodies recognize epitopes on p65, p50, p52, RelB and RelC proteins upon DNA binding (Guglielmotto et al., 2012).

### Statistical Analysis

Statistical analyses were performed using GraphPad Prism version 4.0 (GraphPad software, San Diego). All values were presented as mean ± standard error (SEM). Means were compared by one or two-way analysis of variance (ANOVA) with Bonferroni as a *post hoc* test (Manassero et al., 2016).

## Results

### The Uch-L1 Activity Decrease Is Common Event in AD and VD

**Figure 1:**
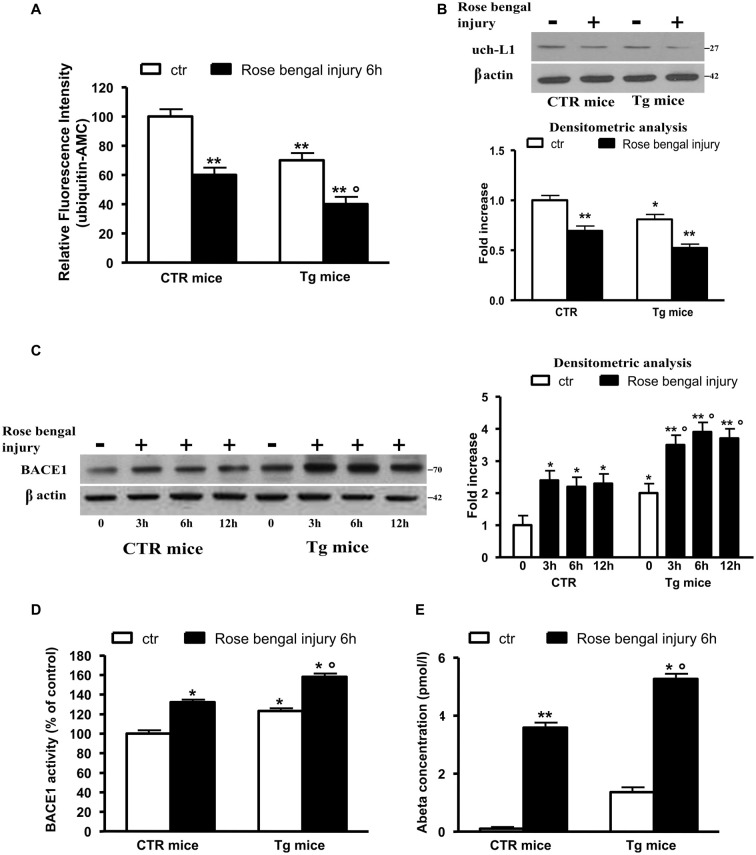
Ubiquitin C-terminal hydrolase L1 (Uch-L1) decrease mediates BACE1 up-regulation. **(A)** Hydrolase activity in control and Tg mice subjected or not to Rose Bengal photo-activation and sacrificed 6 h later. Hydrolase activity was significantly decreased in control mice and further inhibited in Tg mice. **(B)** Representative western blot of brain extracts from control and Tg mice subjected or not to Rose Bengal photo-activation using Uch-L1 antibody for detection. β actin served as loading control. Densitometric quantification shows that Tg mice have slightly lower basal protein levels respect to controls but after thrombotic ischemia the Uch-L1 levels are inhibited resulting significant not only in Tg controls but also respect to ischemic injured control mice. **(C)** Representative western blot of brain extracts from control and Tg mice subjected or not to Rose Bengal photo-activation using BACE1 antibody for detection. β actin served as loading control. Densitometric quantification shows that Tg mice have higher basal protein levels respect to controls but after thrombotic ischemia the BACE1 levels are increased resulting significant not only in Tg controls but also respect to ischemic injured control mice. **(D)** BACE1 activity in control and Tg mice subjected or not to Rose Bengal photo-activation. BACE1 activity was significantly increased in control mice and further enhanced in Tg mice. **(E)** Aβ 42 concentration in brain extracts of control and Tg mice subjected or not to Rose Bengal photo-activation. The vascular injury in wild type mice was followed by a significant increase in Aβ 42 levels with respect to controls. The basal levels of Aβ 42 in Tg mice were significantly higher than those of both control mice subjected or not to photothrombosis. The ischemic injury in Tg mice was followed by a further significant production of Aβ 42. The data are mean ± standard error (SEM). *p < 0.05 vs. control mice; **p < 0.01 vs. control mice; °*p* < 0.05 vs. control mice subjected to Rose Bengal photo-activation. *N* = 6.

We performed all experiments in control or 5xFAD Tg mice subjected or not to thrombotic focal cerebral ischemia mediated by photo-activation with Rose Bengal and then sacrificed up to 12 h later. Figure 1A reports the hydrolase activity; as shown, the thrombotic ischemia in control mice was followed by approximately 40% decrease of activity, whereas the ischemic injury induced a drastic decrease (−70%) of hydrolase levels in Tg mice that presented significant lower basal levels of the enzyme (−30%).

To confirm the role of Uch-L1 in the decreased hydrolase levels we performed western blot analysis using monoclonal Uch-L1 antibody and we found a significant decrease in the enzyme protein levels in both controls and Tg mice after ischemic injury (Figure 1B). We previously reported that the decrease of Uch-L1 activity corresponded to an increase in BACE1 protein levels (Guglielmotto et al., 2012). Here we confirmed that the two events are related since the vascular injury was able to significantly (approximately 2.5-fold) increase BACE1 protein levels. Tg mice have higher basal protein levels respect to controls (2-fold) but after thrombotic ischemia the BACE1 levels are increased, resulting significant not only vs. Tg controls but also respect to ischemic injured control mice (2-fold increase vs. Tg controls and 3-fold increase vs. controls ischemic injured; Figure 1C). To confirm that BACE1 was active, we measured the enzymatic activity and we confirmed that vascular injury increased BACE1 activity of 20% in control mice whereas in Tg mice produced a 50% increase; the basal activity of BACE1 in Tg mice was increased respect to control mice of approximately 25% (Figure 1D). We also measured levels of Aβ 42 in our experimental models (Figure 1E). The vascular injury in wild type mice was followed by a significant increase in Aβ 42 levels respect to control animals in which the levels were almost undetectable. As expected, the basal levels of Aβ 42 in Tg mice were significantly higher than those of control mice subjected or not to photothrombosis. The ischemic injury in Tg mice was followed by a further significant production of Aβ 42 (Figure 1D).

Thus, we observed that the decrease in Uch-L1 determined an increase of BACE1 protein levels and activity and an increased production of Aβ 42. Moreover, we can affirm that the decrease of Uch-L1 is a common event in AD and VD and that when both pathologies are present there is a cumulative effect.

### The Decrease of Uch-L1 Depends on the Induction of NF-κB

**Figure 2:**
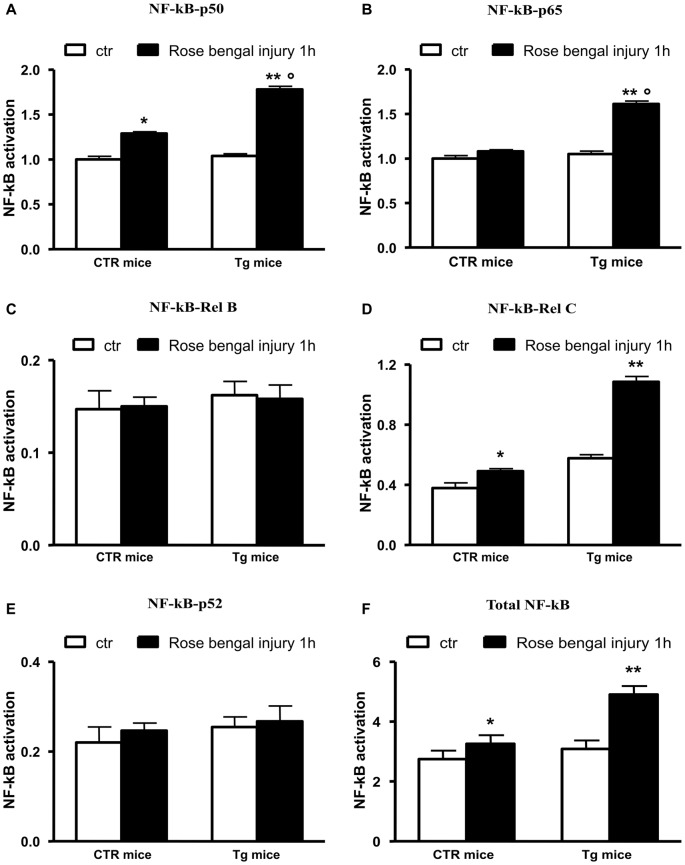
NF-κB pathway is activated in control as well as Tg mice exposed to Rose Bengal photo-activation. The total NF-κB activation **(F)** has been evaluated by screening all members of NF-κB family **(A–E)**. The ischemic injury was followed by a nuclear activation of p50 and p65, RelC in Tg mice. In control mice p50 did not seem activated. The data are mean ± standard error (SEM). *p < 0.05 vs. control mice; **p < 0.01 vs. control mice; °*p* < 0.05 vs. control mice subjected to Rose Bengal photo-activation. *N* = 6.

We studied this pathway because it had been previously reported that Aβ 42 regulates BACE1 promoter transactivation and activity through NF-κB pathway (Buggia-Prevot et al., 2008) and that the activation of this pathway abolishes Uch-L1 gene transcription (Wang et al., 2011). We also previously found that the pharmacological inhibition of NF-κB by blocking the nuclear translocation of p50 or p65 in an *in vitro* model was followed by a completely protection of the Uch-L1 decrease as well as of the BACE1 increase (Guglielmotto et al., 2012). Thus, we investigated whether the focal ischemia could activate NF-κB in our experimental model. We demonstrated that the ischemic injury was able to induce nuclear activation of total NF-κB obtained by screening all NF-κB family members (Figure 2) in control mice and Tg mice exposed to Rose Bengal photo-activation. The results suggest that this pathway could be responsible for the decrease of Uch-L1.

### The Decrease of Uch-L1 Is Followed by Necrotic and Apoptotic Cell Death

**Figure 3:**
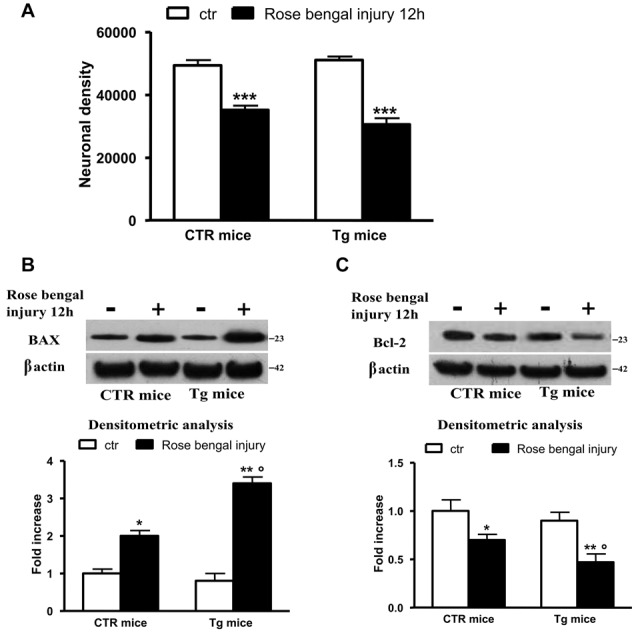
The decrease of Uch-L1 was followed by cell death. **(A)** The neuronal density after lesion was significant decreased in control and Tg mice exposed to Rose Bengal photo-activation respect to not lesioned mice. **(B,C)** Representative western blot of brain extracts from control and Tg mice subjected or not to Rose Bengal photo-activation using Bax **(B)** and Bcl-2 **(C)** antibodies. β actin served as loading control. Densitometric quantification shows that the ischemic injury caused a significant increase of BAX protein and a parallel significant decrease of Bcl-2 in control mice. The ischemic injury induced in Tg mice was followed by a further increase in Bax and decrease in Bcl-2 levels, that resulted significant respect to lesioned control mice. The data are mean ± standard error (SEM). *p < 0.05 vs. control mice; **p < 0.01 vs. control mice; ***p < 0.01 vs. control mice; °*p* < 0.05 vs. control mice subjected to Rose Bengal photo-activation. *N* = 6.

We evaluated the neuronal density after lesion and we found that there was a significant decrease in control and Tg mice exposed to Rose Bengal photo-activation respect to not lesioned mice (Figure 3A). To determine the amount of apoptotic cell death we measured the protein levels of the proapoptotic effector BAX as well as the antiapoptotic protein Bcl-2. As reported in Figures 3B,C, the focal ischemia caused a significant increase of BAX protein (2-fold) and a parallel significant decrease in Bcl-2 (40%) in control mice. The ischemic injury induced in Tg mice was followed by a further increase in Bax (3.5-fold) and decrease in Bcl-2 (−60%) levels, that resulted significant respect to lesioned control mice (Figures 3B,C).

### Restoration of Uch-L1 Corrects the BACE1 Induction and Prevents Cell Death

To foster the levels of Uch-L1 we injected control and Tg mice with a fusion protein between transduction domain of the HIV-transactivator protein (TAT), fused with an HA tag, and Uch-L1 (TAT-HA-Uch-L1; Gong et al., 2006) 30 min before ischemic injury.

**Figure 4:**
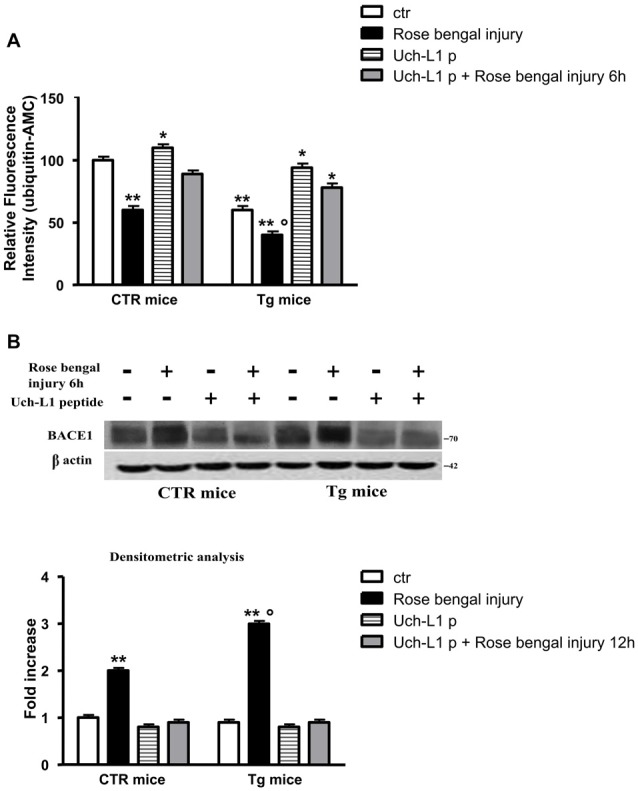
Uch-L1 restoration counteracts the BACE1 up-regulation. **(A)** Hydrolase activity in control and Tg mice subjected or not to Rose Bengal photo-activation and sacrificed 6 h later. The injection of TAT-HA-Uch-L1 re-established normal hydrolase activity both in control as well as in Tg mice exposed to photoischemic injury. **(B)** Representative western blot of brain extracts from control and Tg mice subjected or not to Rose Bengal photo-activation using BACE1 antibody for detection. β actin served as loading control. Densitometric quantification shows that the restoration of Uch-L1 was able to completely prevent the increase of BACE1 protein levels both in control and Tg mice at 6 h post injury. The data are mean ± standard error (SEM). *p < 0.05 vs. control mice; **p < 0.01 vs. control mice; °*p* < 0.05 vs. control mice subjected to Rose Bengal photo-activation. *N* = 6.

**Figure 5:**
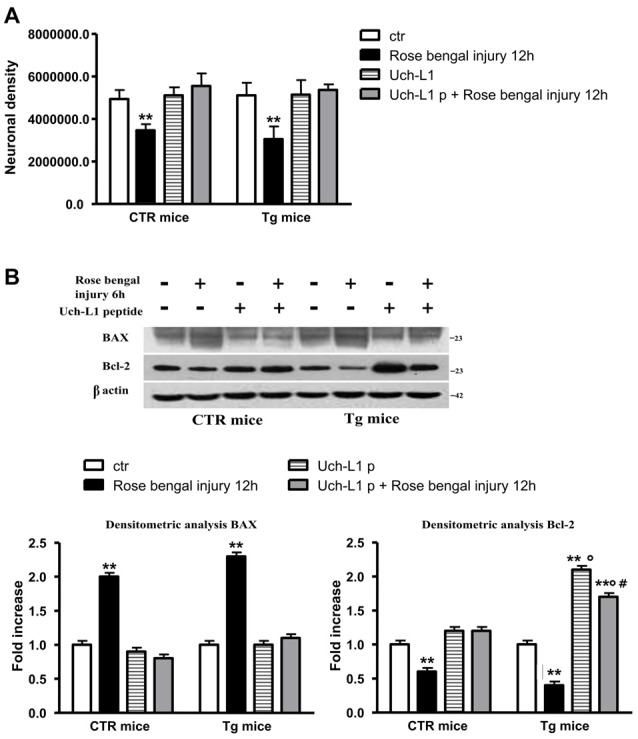
Uch-L1 restoration protects cell death. **(A)** The restoration of Uch-L1 exerts a positive effect on neuronal density in pre-lesioned Tg treated animals respect to untreated lesioned ones. **(B)** Representative western blot of brain extracts from control and Tg mice subjected or not to Rose Bengal photo-activation and pre-treated or not with TAT-HA-Uch-L1 using Bax and Bcl-2 antibodies. β actin served as loading control. Densitometric quantification shows that the restoration of Uch-L1 almost completely prevent the release of pro-apoptotic Bax and the decrease of anti-apoptotic Bcl-2 proteins, both in controls and Tg mice. The data are mean ± standard error (SEM). **p < 0.01 vs. control mice; °*p* < 0.02 vs. Rose Bengal; ^#^*p* < 0.05 Uch-L1p vs. Uch-L1p + Rose Bengal. *N* = 6.

To evaluate if the peptide was able to cross the blood brain barrier we performed a western blot using HA antibody, thus HA is attached to the peptide. A band is visible in cerebral homogenates after treatment with Uch-L1 peptide (data not shown). As reported in Figure 4A, the injection of TAT-HA-Uch-L1 restored normal Uch-L1 activity both in control and in Tg mice exposed to photoischemic injury (Figure 4A). The restoration of Uch-L1 was able to completely prevent the increase of BACE1 protein levels both in control and Tg mice at 6 h post injury, as reported by the representative blot and by the densitometric analysis (Figure 4B). Finally, we also found that pre-treatment with TAT-HA-Uch-L1 was able to protect against the neuronal cell death, indeed we found that the restoration of Uch-L1 significantly increases the percentage of neuronal density in pre-lesioned Tg treated animals respect to untreated lesioned ones (Figure 5A). Finally, we found that the restoration of Uch-L1 rescues Bax control levels (Figure 5B) and protects the decrease Bcl-2, both in controls and Tg mice.

## Discussion

The “vascular hypothesis of AD” is supported by studies showing that vascular pathology leads to inability of Aβ clearance from the brain, producing Aβ accumulation in parenchyma and blood vessels (Canobbio et al., 2015; Janota et al., 2016). Vascular pathology includes macroinfarcts and microinfarcts, atherosclerosis as well as cerebral amyloid angiopathy.

Several studies show vascular pathology in 50% of the elderly population and confirm that the simultaneous presence of vascular and AD lesions is closely related to the severity of dementia (Schneider, 2009; Attems and Jellinger, 2014).

Furthermore, the co-existence of vascular damage and AD appears to be an important condition in determining a further and significant decrease in cognitive capacity with respect to subjects with AD alone (Schneider and Bennett, 2010). Some authors suggested that vascular contribution in AD is much broader that the tissue injury seen in pathologic and imaging studies. Thus, vessel disease and vascular injury are associated with AD but their role in the development of the disease remains unclear.

In this work we found that the focal ischemic microinfarctions induced by laser excitation of the photosensitive dye Bengal Rose determined the decrease of Uch-L1 as well as BACE1 over-expression. We previously demonstrated that Aβ 42 decreased the activity of Uch-L1 by activating NF-κB pathway and that this event up-regulated BACE1 (Guglielmotto et al., 2012). It has been reported that NF-κB pathway transactivates BACE1 promoter (Buggia-Prevot et al., 2008) and concomitantly down-regulates Uch-L1 expression (Wang et al., 2011). Thus, we suggest that, also in this experimental model, the decrease of Uch-L1 and the increase of BACE1 are mediated by NF-κB pathway. Other authors found a down-regulation of Uch-L1 in the hippocampus of AD brain. It has been found that Aβ may down-regulate Uch-L1 in the AD brain, which in turn impairs BDNF/TrkB-mediated retrograde signaling, compromising synaptic plasticity and neuronal survival (Poon et al., 2013). Moreover, a recent study by Öhrfelt et al. (2016) first assessed the potential role of Uch-L1 as a CSF biomarker for AD. Thus, CSF Uch-L1 seems to correlate with CSF total and phospho Tau. Large literature data support the notion that inflammation plays a crucial role in mediating vascular complications and dysfunctions (Ross, 1999; Charo and Taubman, 2004; Hansson and Libby, 2006). Recently, the pro-inflammatory cytokine TNFα has been found a prominent factor in the pathogenesis of vascular diseases (McKellar et al., 2009) and it has been observed that NF-κB cascade is a crucial component of TNFα signal transduction (Bradley, 2008).

In this context, Ichikawa et al. (2010) reported that an up-regulation of Uch-L1 mediates a negative feedback to TNF-α mediated vascular inflammation. Uch-L1 was also found expressed in both human endothelial cells and vascular smooth muscle cells and data obtained by Takami et al. (2007) suggested that Uch-L1 may partially attenuate vascular remodeling through the down-regulation of NF-κB pathway. Specifically, Uch-L1 decreased the NF-κB activity induced by TNF-α and increased eNOS expression, which was able to protect atherosclerosis reducing ischemic vascular disease (Takami et al., 2007). Additionally, the release of prostaglandins, such as biologically active cyclopentenone prostaglandins, that are massively produced in the rat brain after temporary focal ischemia, selectively blocks Uch-L1 activity (Liu et al., 2011). Our current and previous data (Guglielmotto et al., 2012) suggest that the decrease of Uch-L1 is part of a loop that potentiates Aβ accumulation in vascular injury and that the restoration of Uch-L1 activity could represent a novel therapeutic strategy for AD and vascular dementia.

## Author Contributions

MG designed the study, performed the experiments and analyzed the results; DM, VV, IER and GM collaborated in performing the experiments; MT designed the study and wrote and edited the manuscript; ET designed the study and wrote the manuscript.

## Conflict of Interest Statement

The authors declare that the research was conducted in the absence of any commercial or financial relationships that could be construed as a potential conflict of interest.
